# SPILLOVER EFFECTS OF RESIDENTIAL SPATIAL SOCIAL POLARIZATION AND HIGH BLOOD PRESSURE AMONG VA NURSING HOME RESIDENTS

**DOI:** 10.1093/geroni/igad104.0019

**Published:** 2023-12-21

**Authors:** Hoda Abdel Magid, Michelle Odden

**Affiliations:** Stanford University, Stanford, California, United States; Stanford University, Stanford, California, United States

## Abstract

Spatial social polarization (SSP) – the process by which a population within an area diverges across socioeconomic characteristics– has been associated with hypertension. SSP indices measure the extent to which a population is distributed at extremes of privilege and deprivation. The VA nursing home represents an environment where spatially-oriented disparities are expected to be attenuated due to the fact that Veterans have equal access to care. We examined the association between SSP measured at residents’ home addresses prior to admission with blood pressure (BP) outcomes in the first four weeks after admission. We evaluated the use of the index of concentration (ICE) of extremes to measure SSP across four socioeconomic domains including race/ethnicity, income, home ownership, and joint race/ethnicity with income at the tract level using 2010 census data. SSP was dichotomized as the first (most polarized and disadvantaged) quintile vs. the 2nd -5th quintiles. The analytic sample included 41,972 long-term care residents aged ≥65 years admitted from 2006-2019. Multilevel mixed-effects regression models were adjusted for individual demographics and chronic conditions. We found Veterans who had resided in the most polarized and disadvantaged quintile (Q1) had a 1.10 (95% 1.01, 1.19) relative risk of high BP compared to those in Q 2-5 for the ICE jointly measuring race/ethnicity and income. We found similar results for systolic BP. In summary, SSP that jointly measures economic and racial/ethnic polarization, may have a spillover effect on health disparities even in a healthcare setting with equal access.

